# Consumption of NADPH for 2-HG Synthesis Increases Pentose Phosphate Pathway Flux and Sensitizes Cells to Oxidative Stress

**DOI:** 10.1016/j.celrep.2017.12.050

**Published:** 2018-01-09

**Authors:** Susan J. Gelman, Fuad Naser, Nathaniel G. Mahieu, Lisa D. McKenzie, Gavin P. Dunn, Milan G. Chheda, Gary J. Patti

**Affiliations:** 1 Department of Chemistry, Washington University, St. Louis, MO 63130, USA; 2 Department of Medicine, Washington University School of Medicine, St. Louis, MO 63110, USA; 3 Departments of Neurological Surgery and Pathology and Immunology, Washington University School of Medicine, St. Louis, MO 63110, USA; 4 Department of Neurology, Washington University School of Medicine, St. Louis, MO 63110, USA

## Abstract

Gain-of-function mutations in isocitrate dehydroge-nase 1 (*IDH1*) occur in multiple types of human cancer. Here, we show that these mutations significantly disrupt NADPH homeostasis by consuming NADPH for 2-hydroxyglutarate (2-HG) synthesis. Cells respond to 2-HG synthesis, but not exogenous administration of 2-HG, by increasing pentose phosphate pathway (PPP) flux. We show that 2-HG production competes with reductive biosynthesis and the buffering of oxidative stress, processes that also require NADPH. *IDH1* mutants have a decreased capacity to synthesize palmitate and an increased sensitivity to oxidative stress. Our results demonstrate that, even when NADPH is limiting, *IDH1* mutants continue to synthesize 2-HG at the expense of other NADPH-requiring pathways that are essential for cell viability. Thus, rather than attempting to decrease 2-HG synthesis in the clinic, the consumption of NADPH by mutant IDH1 may be exploited as a metabolic weakness that sensitizes tumor cells to ionizing radiation, a commonly used anti-cancer therapy.

## Graphical Abstract

**In Brief:** Using liquid chromatography/mass spectrometry (LC/MS) and stable isotope tracing, Gelman et al. find that 2-HG production in cells with *IDH1* mutations leads to increased pentose phosphate pathway activity to generate NADPH. Production of 2-HG competes with other NADPH-dependent pathways and sensitizes cells to redox stress.

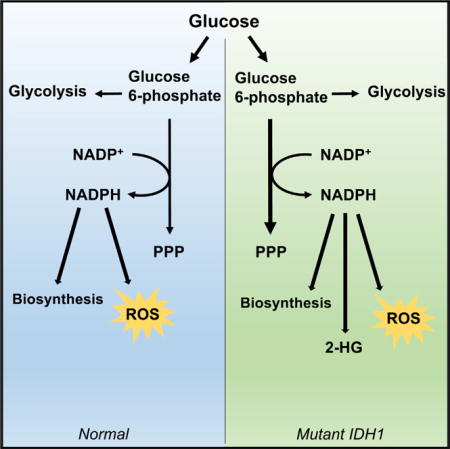

## Introduction

There are three subtypes of isocitrate dehydrogenase (IDH) that vary according to their cellular location and NADP^+^ or NAD^+^ dependency (Dang and Su, 2017). Each subtype oxidatively decarboxylates isocitrate to alpha-ketoglutarate under most normal physiological conditions, but the NADP^+^-dependent IDH1 is uniquely situated in the cytosol, peroxisomes, and endoplasmic reticulum, whereas the NADP^+^-dependent IDH2 and the NAD^+^-dependent IDH3 are in the mitochondrial matrix (Geisbrecht and Gould, 1999; Lewis et al., 2014; Margittai and Bánhegyi, 2008). The cellular location of the IDH1 and IDH2 enzymes is particularly important because NADPH does not have protein transporters to cross the inner mitochondrial membrane (Pollak et al., 2007). However, both the cytosol and the mitochondria have essential NADPH demands. In the cytosol, NADPH is needed for the reductive biosynthesis of palmitate and cholesterol (Lunt and Vander Heiden, 2011). In the cytosol and the mitochondria, NADPH is a required cofactor for the glutathione and thioredoxin systems to neutralize reactive oxygen species that result from oxidative stress (Ren et al., 2017). Thus, each cellular compartment must independently balance their NADPH production and consumption rates.

In this study, we were interested in assessing changes in NADPH homeostasis that result from a mutation in *IDH1* substituting an arginine for a histidine at codon 132 (R132H). This *IDH1* R132H mutation is prevalent in several forms of human cancer, such as low-grade gliomas and secondary glioblastomas (Cohen et al., 2013; Yan et al., 2009). Importantly, not only does the R132H mutation result in the loss of the enzyme’s ability to produce NADPH, it also confers a gain of enzyme function that consumes NADPH (Dang et al., 2009). Wild-type IDH1 reduces NADP^+^ to NADPH while converting isocitrate to alpha-ketoglutarate. Mutant IDH1, on the other hand, oxidizes NADPH to NADP^+^ while converting alpha-ketoglutarate to the metabolite 2-hydroxyglutarate (2-HG). Notably, 2-HG accumulates to millimolar concentrations in the media of *IDH1* mutant cells as well as in some *IDH1* mutant tumors (Dang et al., 2009), suggesting that its synthesis requires a large amount of NADPH. Here, we sought to determine the effect that this metabolic demand for NADPH has on other NADPH-requiring pathways, particularly when NADPH is limiting.

We considered two potential metabolic consequences of the NADPH demands imposed by 2-HG synthesis. One possibility is that consuming NADPH for 2-HG synthesis results in a shortage of NADPH. Indeed, it has been speculated that using NADPH for 2-HG synthesis further contributes to an NADPH deficit due to impaired wild-type IDH1 activity, which is a major source of NADPH in some cells (Atai et al., 2011; Losman and Kaelin, 2013). We predicted that such a deficit in NADPH could limit the activity of other NADPH-requiring reactions, such as those involved in reductive biosynthesis and the buffering of oxidative stress. An alternative possibility is that cells increase their production of NADPH to compensate for impaired IDH1 wild-type activity and 2-HG synthesis. Directing more glucose carbon through the pentose phosphate pathway (PPP), for example, allows for increased production of NADPH.

In this work, we evaluated HCT116 human colorectal carcinoma cells with a knockin heterozygous R132H mutation at the *IDH1* locus. Given that tumor-associated *IDH* mutations are usually observed to occur in the heterozygous state in the clinic, these cells mimic those found in the tumors of patients. We also extended the scope of our study by using immortalized human astrocytes with transgenic *IDH1* R132H, which displayed a comparable metabolic phenotype. We found that although both of these cell lines do increase their production of NADPH by the PPP to support 2-HG synthesis, the NADPH produced is insufficient for all NADPH-requiring reactions, particularly under conditions of oxidative stress. Reductive biosynthesis, glutathione reductase, and 2-HG synthesis therefore cannot all be adequately supported. Interestingly, cells continue to synthesize 2-HG even though it directs NADPH away from other reactions that are required for cell viability.

## Results

### *IDH1* Mutants Can Synthesize 2-HG from Glucose or Glutamine Carbon

**Figure 1:**
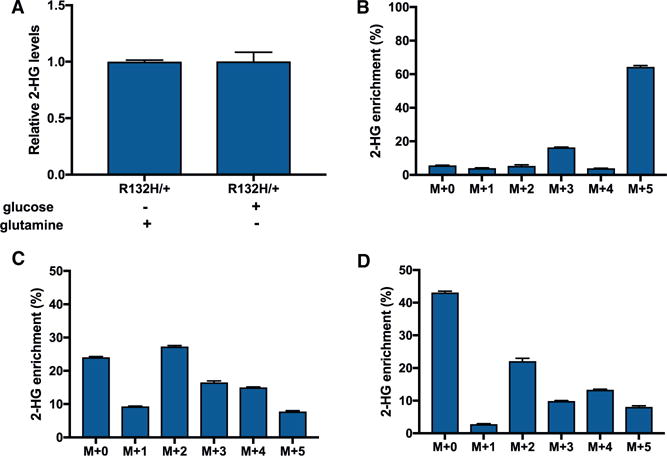
Carbon for 2-HG Synthesis Can Be Derived from Glucose or Glutamine (A) Relative level of 2-HG in wild-type HCT116 cells grown with 25 mM glucose and no glutamine (other than that from FBS), or 4.5 mM glutamine and no glucose (other than that from FBS). Irrespective of condition, the cells synthesized 2-HG. (B) Isotopologue distribution pattern of 2-HG after HCT116 R132H/+ cells were labeled with U-^13^C glutamine, with no glucose (other than that from FBS) present in the culture media. (C) Isotopologue distribution pattern of 2-HG after HCT116 R132H/+ cells were labeled with U-^13^C glucose, with no glutamine (other than that from FBS) present in the culture media. (D) Isotopologue distribution pattern of 2-HG after HCT116 R132H/+ cells were labeled with U-^13^C glucose in the presence of 4.5 mM glutamine. Data shown are mean values ± SD (n = 3).

We aimed to understand the metabolic flexibility that *IDH1* mutant cells have in synthesizing 2-HG. We first considered the carbon source from which 2-HG is synthesized. Although glutamine has been primarily considered as the major precursor to 2-HG (Dang et al., 2009), we found that 2-HG can also be produced from glucose. When HCT116 cells were cultured in media without glutamine, they still produced 2-HG at high levels (Figure 1A). Moreover, experiments tracking uniformly labeled ^13^C glutamine (U-^13^C glutamine) or uniformly labeled ^13^C glucose (U-^13^C glucose) showed that both are used as a source of 2-HG carbon (Figures 1B–1D). These data suggest that *IDH1* mutants have flexibility with respect to the carbon source of 2-HG and are consistent with previous reports (Izquierdo-Garcia et al., 2015).

### Increased PPP Flux in *IDH1* Mutants

**Figure 2:**
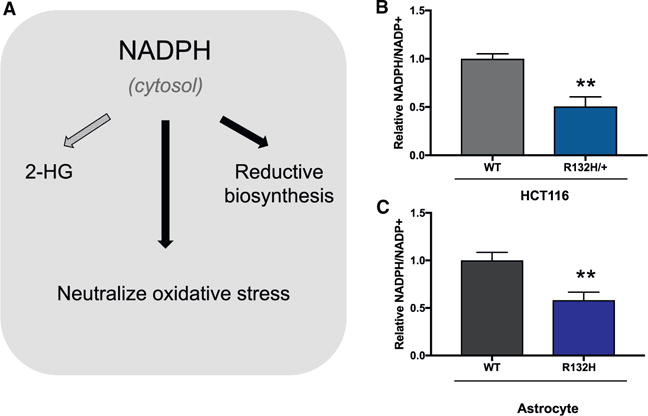
Evaluating NADPH in *IDH1* Mutant Cells (A) In the cytosol, NADPH is required for 2-HG synthesis, for reductive biosynthesis (e.g., fatty acid synthesis), and to neutralize reactive oxygen species (e.g., by the glutathione system). (B and C) The ratio of NADPH to NADP^+^ decreases in HCT116 (B) and astrocyte R132H (C) mutants compared to wild-type (WT) cells. Ratios were determined by using a colorimetric assay. Data shown are mean values ± SD (n = 3). **p value < 0.01.

**Figure 3:**
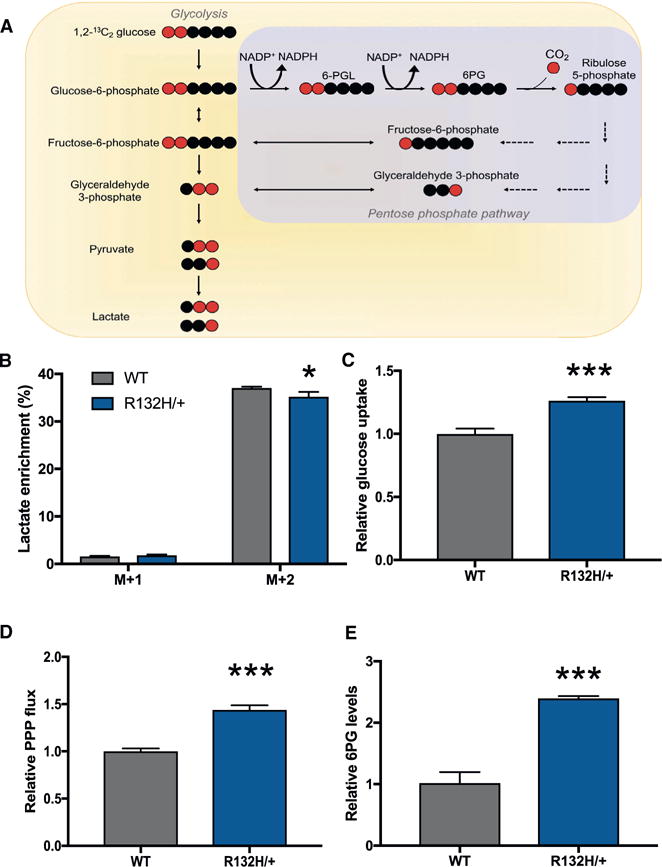
IDH1 Mutants Have Increased PPP Flux (A) Schematic showing lactate labeling when 1,2-^13^C_2_ glucose is metabolized through glycolysis directly, and when 1,2-^13^C_2_ glucose is metabolized through the PPP and then fed back into glycolysis. Red circles correspond to ^13^C-labeled carbons, and black circles correspond to unlabeled ^12^C carbons. (B) Isotopologue distribution of lactate in wild-type cells and HCT116 R132H/+. The M+1 isotopologue is a result of glucose that passed through the PPP. The M+2 isotopologue corresponds to glucose that was metabolized to lactate through glycolysis directly. (C) Uptake of glucose by wild-type cells and mutant *IDH1* as measured by LC/MS analysis of the media. (D) Relative PPP flux, as determined by lactate labeling from 1,2-^13^C_2_ glucose and glucose uptake. (E) The PPP intermediate 6-phosphogluconate (6PG) is increased in *IDH1* mutants relative to WT cells. Data shown are mean values ± SD (n = 3). *p value < 0.05; ***p value < 0.001.

Given the observed flexibility in carbon for 2-HG synthesis, we turned our attention to NADPH as a potential limiting factor (Figure 2A). Not only is NADPH required to synthesize 2-HG from alpha-ketoglutarate, *IDH1* mutants also have reduced NADPH production due to impaired wild-type IDH1 activity (Bleeker et al., 2010). For both HCT116 cells and astrocytes, we measured a significantly decreased NADPH/NADP^+^ ratio in *IDH1* mutants relative to wild-type controls (Figures 2B and 2C). We next set out to determine whether cells responded to this altered ratio by increasing NADPH production. Since IDH1 is localized to the cytosol, we focused on the PPP, which is a major source of cytosolic NADPH (Lewis et al., 2014). To assess the flux of the PPP, we first applied a previously established method using liquid chromatography/mass spectrometry (LC/MS) to trace ^13^C labels from 1,2-^13^C_2_ glucose (Lee et al., 1998; Li et al., 2014). When 1,2-^13^C_2_ glucose is metabolized directly through glycolysis, without entering the PPP, lactate containing two ^13^C labels is produced. When 1,2-^13^C_2_ glucose is metabolized through the PPP, in contrast, an oxidative decarboxylation reaction removes the ^13^C label on the first position of glucose. This produces ribulose 5-phosphate containing one ^13^C label, which then can be inserted back into glycolysis via the non-oxidative reactions of the PPP. The result is lactate containing only a single ^13^C label (Figure 3A). The ratio of singly labeled lactate (the M+1 isotopologue) to doubly labeled lactate (the M+2 isotopologue) represents the ratio of flux through the PPP to flux directly through glycolysis.

We found a statistically significant difference in lactate labeling from wild-type HCT116 control cells and *IDH1* mutants (Figure 3B). By media analysis, we determined that *IDH1* mutants have a significantly higher rate of glucose uptake relative to wild-type controls (Figure 3C). We then calculated PPP flux by using the difference in lactate labeling normalized by glucose uptake. We found that the *IDH1* mutants have a 40% increase in PPP flux relative to wild-type controls (Figure 3D; Table S1). These data are consistent with the 40% increase measured in the concentration of the PPP intermediate 6-phosphogluconate (6PG) in *IDH1* mutants relative to wild-type cells (Figure 3E) and puts the PPP flux in the same order of magnitude as the rate of 2-HG synthesis (Table S1). We also note that increased PPP flux in *IDH1* mutants relative to wild-type cells was determined to be independent of fetal bovine serum (FBS) concentration (Figures S1A–S1C).

A limitation of using the lactate-labeling method above to assess PPP flux is that it is not specific to the oxidative phase of the pathway, which is where NADPH is produced. One possibility is that ribulose 5-phosphate produced by the oxidative phase of the PPP does not re-enter glycolysis to become lactate. In some cancers, for example, the oxidative phase and the non-oxidative phase of the PPP may run in the same direction toward ribose 5-phosphate production (Liu et al., 2010). To specifically assess the rate of NADPH production by the oxidative phase of the PPP, we performed kinetic flux profiling as has been described previously (Yuan et al., 2008). Cells were given U-^13^C glucose and incorporation of label from glucose 6-phosphate into 6PG was measured as function of a time (Figures S1D and S1E). Data from kinetic flux profiling were consistent with the data from lactate labeling above, showing an 44% increase in flux of the oxidative PPP in *IDH1* mutants relative to wild-type cells.

### Assessing Malic Enzyme Flux in *IDH1* Mutants

Classically, the oxidative PPP is thought to be the primary source of cytosolic NADPH (Fan et al., 2014). The malic enzyme (ME), however, also produces NADPH, and therefore we sought to evaluate whether ME flux increased in *IDH1* mutants. Cells were given U-^13^C glutamine, and lactate labeling, which represents ME activity, was compared between wild-type cells and *IDH1* mutants (Figure S2A). Although our analysis cannot distinguish between the cytosolic and mitochondrial subtypes of ME, the data did not support an overall increase in ME activity in *IDH1* mutants (Figure S2B).

### Inhibiting 2-HG Synthesis Reduces PPP Flux

**Figure 4:**
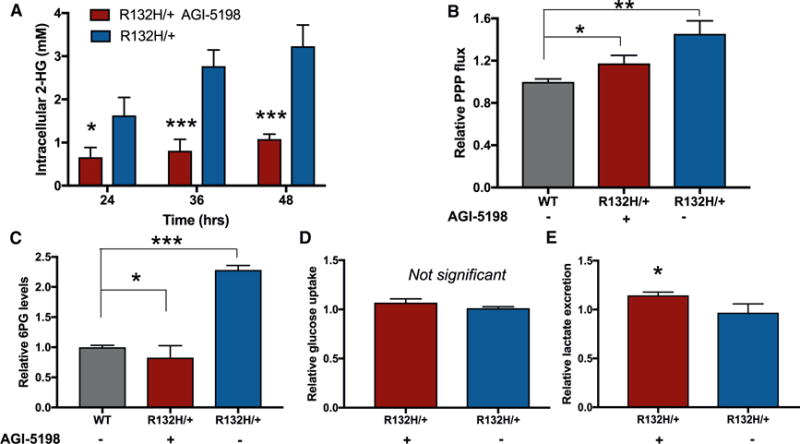
Effects of AGI-5198 on 2-HG Synthesis, PPP Flux, and Glucose Uptake (A) AGI-5198 effectively inhibits 2-HG synthesis. HCT116 R132H/+ cells were exposed to either AGI-5198 or vehicle (DMSO) for 72 hr prior to making the intracellular measurements 24, 36, or 48 hr later. (B) Relative PPP flux in untreated wild-type control cells, *IDH1* mutants treated with AGI-5198, and untreated *IDH1* mutants. (C) Intracellular levels of the PPP intermediate 6PG are consistent with changes observed in PPP flux. (D) Uptake of glucose from the media is not significantly altered in *IDH1* mutants due to AGI-5198 treatment. (E) Lactate excretion increases when *IDH1* mutants are treated with AGI-5198. Data shown are mean values ± SD (n = 3). *p value < 0.05, **p value < 0.01, and ***p value < 0.001.

To test our hypothesis that PPP flux increases to produce NADPH in support of 2-HG synthesis, we treated *IDH1* mutants with an inhibitor of 2-HG and measured changes in PPP activity. We pharmacologically inactivated R132H-IDH1 with the selective inhibitor AGI-5198 (Rohle et al., 2013), which we found to decrease intracellular 2-HG levels by 70% (Figure 4A). We exposed cells to 0.2 mM AGI-5198 (dissolved in DMSO) for 72 hr prior to labeling with 1,2-^13^C_2_ glucose. By applying the same method as above, we measured PPP flux. Although AGI-5198 treatment did not completely restore PPP flux to the level of wild-type cells, it did reduce it significantly (Figure 4B). These results were consistent with the relative changes in 6PG concentration (Figure 4C). Interestingly, glucose uptake was not reduced by AGI-5198 treatment (Figure 4D). Since the additional glucose taken up by *IDH1* mutants relative to wild-type cells was not being directed to the PPP upon AGI-5198 treatment, less of the carbon was being lost as carbon dioxide due to 6PG dehydrogenase activity. Increased glucose flux through the oxidative PPP has been shown to decrease lactate excretion (Zhao et al., 2009), and we therefore speculated that AGI-5198 would increase lactate excretion. Indeed, we measured increased lactate excretion in cells treated with AGI-5198 (Figure 4E).

### Increased PPP Flux Supports the NADPH Demands of 2-HG Synthesis

We next set out to determine whether the increased PPP flux in *IDH1* mutants is to support the NADPH demands of 2-HG synthesis, or whether increased PPP flux is a response to oxidative stress caused by the presence of 2-HG. Previous studies have shown that 2-HG is sufficient in itself to induce oxidative stress (Latini et al., 2003). Thus, to assess potential changes in PPP flux due to oxidative stress associated with the presence of 2-HG, we exposed wild-type cells to 0.1 mM octyl 2-HG for 72 hr prior to providing them with 1,2-^13^C_2_ glucose. We used the octyl ester of 2-HG to improve cell permeability (Xu et al., 2011). The concentration of octyl 2-HG used resulted in intracellular concentrations of 2-HG that are comparable to those measured in *IDH1* mutants (Figure S3A). No statistically significant change in PPP flux was measured as a result of octyl 2-HG treatment (Figure S3B). These data suggest that the changes observed in *IDH1* mutant cells are to support the NADPH demands of 2-HG synthesis, and not to neutralize oxidative stress induced by the presence of 2-HG.

It is provocative to consider blocking the production of NADPH by the PPP as a means to inhibit 2-HG synthesis in *IDH1* mutants; however, the accumulation of PPP intermediates negatively regulates glycolysis (Kahana et al., 1960; Parr, 1956). Cells treated with 6-aminonicotinamide, an inhibitor of 6PG dehydrogenase (Tsouko et al., 2014), do show decreased levels of intracellular 2-HG (Figure S4A). However, interpreting the cause of the reduced intra cellular 2-HG concentration is complicated because these cells also have decreased rates of glycolysis and tricarboxylic acid (TCA) cycle activity (Figure S4B).

### Expression of Glucose 6-Phosphate Dehydrogenase Is Not Significantly Increased in *IDH1* Mutants

Under physiological conditions, glucose 6-phosphate dehydrogenase (G6PD) catalyzes the rate-limiting step of the oxidative PPP and its activity is therefore tightly regulated as a control point (Jiang et al., 2014). Given that NADPH competes with NADP^+^ in binding G6PD, the NADPH/NADP^+^ ratio is a major modulator of enzyme activity (Berg et al., 2002). A decreased NADPH/NADP^+^ ratio (as measured in our *IDH1* mutants; Figure 2) is sufficient to activate G6PD activity (Eggleston and Krebs, 1974; Jiang et al., 2011). G6PD activity has also been shown to be regulated by expression (Patra and Hay, 2014), and we therefore performed qPCR to examine its levels. Our analyses did not reveal a statistically significant difference in expression levels between wild-type and mutant cells (Figure S5A). We note that a high NADPH/NADP^+^ ratio has been reported to result in low enzyme activity (Eggleston and Krebs, 1974), independent of expression levels (Patra and Hay, 2014). Thus, although we did not observe increased expression of G6PD and additional post-translational mechanisms might be at work (Rao et al., 2015), our data indicate that allosteric control is increasing PPP activity in the *IDH1* mutants that we examined.

### Fatty Acid Synthesis Is Decreased in *IDH1* Mutants

Fatty acid synthesis primarily occurs in the cytosol, where acetyl-CoA units are used to make palmitate (Menendez and Lupu, 2007). Other fatty acids are then derived from palmitate through elongation and desaturation reactions (Cook and McMaster, 2002). For each molecule of palmitate synthesized, 14 molecules of NADPH are required (Vander Heiden et al., 2009). Palmitate synthesis is important in rapidly proliferating cancer cells, such as those studied here, to support the formation of new membranes (Yao et al., 2016a).

**Figure 5:**
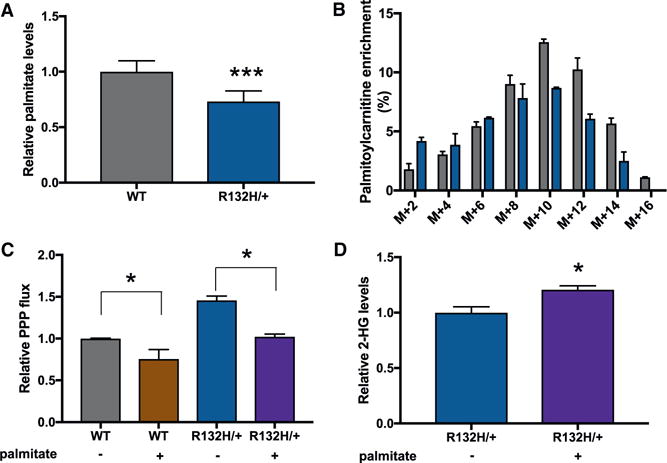
Synthesis of 2-HG and Palmitate Compete for NADPH (A) Relative palmitate levels in wild-type HCT116 cells and R132H/+ mutants. (B) Isotopologue distribution of palmitoylcarnitine in HCT116 wild-type and R132H/+ cells after U-^13^C glucose labeling. (C) Providing exogenous palmitate lowers PPP flux in both R132H/+ and WT HCT116 cells. (D) Relative 2-HG levels in *IDH1* mutants cultured with and without extracellular palmitate. 2-HG is significantly elevated when exogenous palmitate is provided. Data shown are mean values ± SD (n = 3). *p value < 0.05; ***p value < 0.001.

In *IDH1* mutants, we found that the ratio of NADPH to NADP^+^ was significantly reduced (Figures 2B and 2C). This led us to hypothesize that the NADPH-dependent synthesis of palmitate may be limited. We first compared the relative concentration of palmitate and determined that it was decreased in *IDH1* mutants with respect to wild-type controls (Figure 5A). Because standard media contains relatively little palmitate, we associated this difference with a change in palmitate synthesis (Yao et al., 2016a). To more directly test whether the rate of palmitate synthesis was altered, we cultured cells in U-^13^C glucose for 24 hr and used LC/MS to measure the labeling pattern of palmitoylcarnitine. Inferring the palmitate labeling pattern from palmitoylcarnitine has been shown to be consistent with analysis of palmitate directly, but analysis of palmitoylcarnitine is unaffected by potential palmitate contamination introduced during sample handling (Yao et al., 2016b). When comparing *IDH1* mutants to wild-type controls, we observed a decrease in the total enrichment of palmitoylcarnitine (Figure 5B). Additionally, we found that the overall isotopic distribution was shifted toward lighter *m/z* in *IDH1* mutants. To quantitate these differences, we per formed isotopomer spectral analysis (ISA) by using the convISA algorithm implemented in MATLAB (Tredwell and Keun, 2015). *D*_glucose_ represents the fractional enrichment of acetyl-CoA from glucose, and *g*(*t*) represents the fractional *de novo* synthesis of palmitate during 24 or 120 hr of glucose labeling (Table 1). Although *D*_glucose_ was approximately the same between *IDH1* mutants and wild-type cells, the fractional *de novo* synthesis of palmitate decreased by ~20% in *IDH1* mutants. ISA data from 120 hr of labeling, which approached isotopic steady state, suggest that differences in the fractional *de novo* synthesis of palmitate are not a result of differences in proliferation rates and indicate that palmitoylcarnitine labeling is decreased in *IDH1* mutants due to decreased palmitate synthesis.

Given that rapidly proliferating cells may prefer to uptake fatty acids from the media rather than synthesize them *de novo* (Yao et al., 2016a), we next tested whether we could alleviate the NADPH burden due to fatty acid synthesis by providing cells with an exogenous source of palmitate. Cells were first grown in media supplemented with low concentrations of palmitate (40 mM) conjugated to BSA for 24 hr. Cells were then transferred to media with the same formulation, but natural-abundance glucose was replaced with 1,2-^13^C_2_ glucose. After 12 hr of labeling, we calculated PPP flux by applying the same method described above. We found that the addition of exogenous palmitate to the media reduced PPP flux in both wild-type cells and *IDH1* mutants. However, even when cultured in media containing exogenous palmitate, PPP flux was higher in *IDH1* mutants relative to wild-type controls (Figure 5C). Additionally, we found that supplementing HCT116 *IDH1* mutants with extracellular palmitate led to the production of 20% more 2-HG (Figure 5D). This result suggests that decreased palmitate synthesis due to uptake from the media makes more NADPH available for 2-HG synthesis.

### Continued Production of 2-HG Sensitizes Cells to Oxidative Stress

Next, we aimed to test the effect of 2-HG synthesis on the buffering of oxidative stress, which also relies on NADPH. Given that 2-HG synthesis affects NADPH availability, we hypothesized that it may indirectly limit the ability of a cell to neutralize an oxidative insult such as hydrogen peroxide (H_2_O_2_) or ionizing radiation (IR). Both H_2_O_2_ and IR result in highly reactive free radicals that damage proteins and DNA (Azzam et al., 2012; Kuehne et al., 2015). One mechanism to neutralize these reactive oxygen species is the NADPH-dependent glutathione and thioredoxin systems. Since the glutathione and thioredoxin systems are essential to cell survival upon oxidative stress, we were interested whether *IDH1* mutants would prioritize this NADPH demand in the presence of H_2_O_2_ or IR over the NADPH demand of 2-HG synthesis, which is not essential for cell viability.

**Figure 6:**
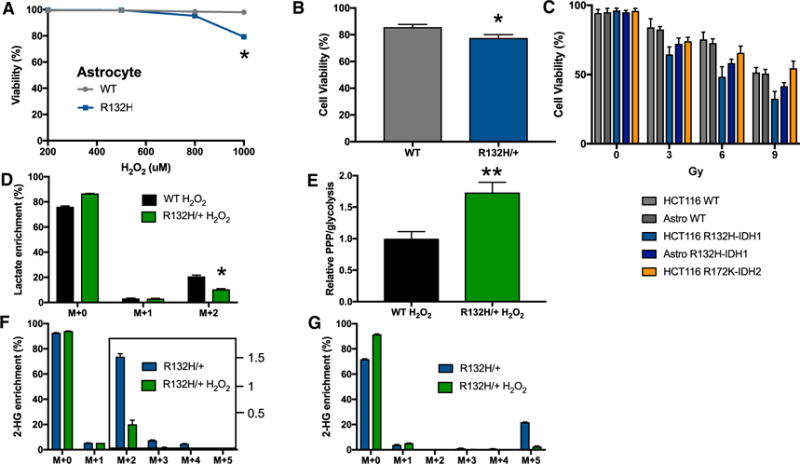
Effects of Oxidative Stress on *IDH1* and *IDH2* Mutant Cells (A) Cell viability as measured by release of LDH from wild-type or *IDH1* mutant astrocytes after H_2_O_2_ treatment for 3 hr at the concentrations shown. (B) Cell viability as measured by release of LDH from wild-type or R132H/+ HCT116 cells after 1 mM H_2_O_2_ treatment for 3 hr. (C) Percent cell viability after 0–9 Gy of IR treatment. Cell viability was measured 72 hr after exposure in HCT116 cells and 96 hr after exposure in astrocytes (astrocytes did not exhibit signs of cell death at 72 hr). (D) Isotopologue distribution of lactate after HCT116 R132H/+ cells were incubated in 1,2-^13^C_2_ glucose and 1 mM H_2_O_2_ for 1 hr. (E) The ratio of PPP to glycolysis is elevated in wild-type HCT116 cells relative to R132H/+ mutants after exposure to H_2_O_2_ for 1 hr. (F) Isotopologue distribution of 2-HG after HCT116 R132/+ cells were incubated in U-^13^C glucose with or without 1 mM H_2_O_2_ for 3 hr. (G) Isotopologue distribution of 2-HG after HCT116 R132H/+ cells were incubated in U-^13^C glutamine, with or without 1 mM H_2_O_2_ for 3 hr. Data shown are mean values ± SD (n = 3). *p value < 0.05; **p value < 0.01.

We first induced oxidative stress by treating *IDH1* mutants or control cells with various concentrations of H_2_O_2_ and then measured cell viability 3 hr later. We found that both HCT116 and astrocyte *IDH1* mutants were more sensitive to 1 mM H_2_O_2_ relative to their wild-type controls (Figures 6A and 6B). As predicted, inhibiting 2-HG synthesis with AGI-5198 improved cell viability in the presence of H_2_O_2_ (Figure S5B).

We then repeated a similar analysis using radiation exposure as the oxidative insult. We treated wild-type cells and *IDH1* mutants with 0, 3, 6, or 9 Gy of IR and measured cell death 72 or 96 hr after exposure (Figure 6C). Consistent with our H_2_O_2_ results, *IDH1* mutants were significantly more sensitive to IR than wild-type controls. We next extended our analysis to HCT116 cells with a *IDH2* mutation substituting an arginine with a lysine at codon 172 (R172K). Similar to the R132H mutation in *IDH1*, the R172K mutation in *IDH2* results in an enzyme gain of function where alpha-ketoglutarate is transformed into 2-HG with the simultaneous oxidation of NADPH to NADP^+^ (Ward et al., 2010). Unlike IDH1, however, IDH2 is localized to the mitochondrial matrix. *IDH2* mutants also showed a significantly increased sensitivity to IR relative to wild-type HCT116 cells (Figure 6C). Interestingly, the *IDH2* mutants were less sensitive to IR than the *IDH1* mutants.

To evaluate the response of the PPP to oxidative stress, we exposed cells to 1 mM H_2_O_2_ for 1 hr while simultaneously labeling them with 1,2-^13^C_2_ glucose during the same time period. In *IDH1* mutant cells, we observed significantly less M+2 labeling in lactate relative to wild-type cells (Figure 6D). Notably, the ratio of the PPP to glycolysis is significantly larger in the *IDH1* mutants compared to wild types (Figure 6E), suggesting that *IDH1* mutants direct more carbon into the PPP and less carbon directly through glycolysis. To determine the extent to which cells continue to synthesize 2-HG upon oxidative insult, we treated *IDH1* mutants with 1 mM H_2_O_2_ for 3 hr. During this 3-hr period, we simultaneously labeled the cells with U-^13^C glucose. Although 2-HG synthesis from glucose was reduced, it was not discontinued (Figure 6F). We then repeated the experiment using U-^13^C glutamine and obtained similar results (Figure 6G), showing that 2-HG continues to be synthesized from both glucose and glutamine during conditions of oxidative stress. Taken together, our results suggest that the NADPH demand imposed by continued 2-HG synthesis in the face of oxidative stress decreases cell viability. Despite the relative increase in PPP activity, the NADPH produced may be insufficient to support both 2-HG synthesis and the buffering of oxidative stress, thereby leading to cell death. Alternatively, cell death may be due to insufficient flux of carbon through glycolysis as a result of elevated PPP activity. Notwithstanding, the increased NADPH demand from 2-HG synthesis during oxidative insult is detrimental to cell survival.

### *IDH1* Mutants Use More Exogenous Acetate for Palmitate Synthesis Than Wild-Type Cells

Our data above support that consumption of NADPH for 2-HG synthesis limits NADPH available for buffering oxidative stress. We also note that the decrease in PPP flux due to 2-HG inhibition is on the same order of magnitude as the decrease in PPP flux due to palmitate supplementation (Figures 4B and 5C), suggesting that both processes contribute to PPP flux. The K_M_ value for NADPH for R132H/+ has been reported as < 0.4 mM (Pietrak et al., 2011), while the K_M_ value for NADPH for the overall reaction catalyzed by human fatty acid synthase has been reported as 5 ± 1 mM (Carlisle-Moore et al., 2005). These values support that the *IDH1* mutant heterodimer binds NADPH more efficiently than fatty acid synthase at low NADPH concentrations, which are observed in *IDH1* mutants (Figures 2B and 2C). In addition to 2-HG production competing for cytosolic NADPH, we also sought to assess whether 2-HG production may compete with palmitate synthesis for carbon utilization. Accordingly, we cultured wild-type cells and *IDH1* mutants in media containing U-^13^C glucose and 15 mM sodium acetate for 24 hr. We then measured palmitoylcarnitine labeling in each sample and applied ISA (Table S2). We point out that the addition of sodium acetate decreased the proliferation rate of both wild-type cells and *IDH1* mutants, thereby preventing the direct comparison of data in Table 1 to Table S2. Nonetheless, although the values of *D*_glucose_ for wild-type cells and *IDH1* mutants were comparable when cultured in normal media, the value of *D*_glucose_ decreased from 0.62 to 0.57 in the presence of exogenous acetate. These results show that *IDH1* mutants use exogenous carbon as a source of acetyl-CoA to a greater extent than wild-type cells, suggesting that 2-HG synthesis may also impose limitations on carbon availability.

We attempted to apply a similar experimental design to that of acetate above by supplementing cells with exogenous reduced glutathione (GSH). Although, in theory, exogenous GSH should reduce cytosolic NADPH demands, GSH added to media spontaneously oxidized over the time course of our experiments and therefore could not reliably be evaluated.

## Discussion

Glioma patients with mutations in *IDH1* are known to have prolonged survival compared to glioma patients of the same grade with wild-type enzyme (Labussiere et al., 2010; Li et al., 2013; Parsons et al., 2008). These different clinical outcomes have been associated with increased sensitivity of *IDH* mutant tumors to the oxidative stress of cytotoxic therapies (Bleeker et al., 2010; Houillier et al., 2010; Mohrenz et al., 2013; Tran et al., 2014). Although the mechanistic basis of this sensitivity has not been established, substantial evidence suggests that it is related to an NADPH deficit that affects cellular responses to reactive oxygen species. Importantly, when tumors with *IDH1* mutations are heterozygous, wild-type enzyme is unable to efficiently produce NADPH (Zhao et al., 2009). In glioblastoma patients, it has been reported that *IDH1* mutations result in about a 2-fold decrease in NADPH production by wild-type enzyme (Bleeker et al., 2010). Since wild-type IDH1 is an important source of NADPH in healthy brain tissue, it has been proposed that R132H mutations lead to an NADPH deficit due to impaired wild-type enzyme activity (Molenaar et al., 2015). Prior to the current study, the potential effect of the NADPH consumed by mutant *IDH1* during 2-HG synthesis had not been quantitatively evaluated.

Here, we show that the actual synthesis of 2-HG creates a significant NADPH demand that is partially supported by a ~40% increase in PPP activity. When mutant *IDH1* is pharmacologically inactivated by AGI-5198, PPP activity is reduced toward wild-type levels. It has been suggested previously that the NADPH deficit in *IDH1* mutants primarily results from 2-HG inactivating wild-type IDH1 enzyme, thereby preventing its NADPH production (Molenaar et al., 2015). Our data suggest, however, that the consumption of NADPH during 2-HG synthesis creates a significant NADPH demand contributing to the NADPH deficit. When we treat wild-type cells with exogenous 2-HG that accumulates to the same intracellular level as when synthesized endogenously by *IDH1* mutant cells, we do not observe an increase in PPP activity as we do in *IDH1* mutant cells. These results indicate that NADPH homeostasis is disrupted by 2-HG synthesis, independent of any interaction between 2-HG and wild-type IDH1 enzyme.

Our data suggest that 2-HG synthesis is regulated by NADPH availability, which is consistent with what is known about the regulation of the wild-type IDH enzyme (Leonardi et al., 2012; Reitman and Yan, 2010). When we increase NADPH availability by partially relieving the burden of fatty acid synthesis with exogenous palmitate, 2-HG synthesis is increased by ~20%. Similarly, when cells are exposed to oxidative stress, NADPH availability is decreased due to elevated activity of NADPH-requiring antioxidant pathways. In this situation, we observe decreased 2-HG synthesis. Interestingly, although 2-HG synthesis is reduced under conditions of oxidative stress, the anabolic process still consumes NADPH at a time when NADPH is critical to neutralize reactive oxygen species. Even this reduced rate of 2-HG synthesis negatively affects cell survival. Thus, while 2-HG is not essential for cell viability, cells continue to consume NADPH for its synthesis in competition with other NADPH-requiring pathways that are essential for cell viability. Consequently, 2-HG synthesis may represent a metabolic vulnerability that sensitizes cells to IR. Inhibiting 2-HG synthesis while co-administering radiotherapy could therefore lead to a worse clinical outcome than radiotherapy alone.

## Experimental Procedures

### Materials

All liquid chromatography solvents and additives were obtained from Sigma-Aldrich (St. Louis, MO) and Honeywell Burdick & Jackson (Morris-town, NJ). All cell culture media and reagents were purchased from Thermo Fisher (Mountain View, CA) or Sciencell (Carlsbad, CA). H_2_O_2_ was purchased from Sigma-Aldrich (St. Louis, MO). Human colorectal carcinoma (HCT116) cells with a heterozygous knockin of *IDH1* mutant (R132H) were obtained from Horizon Discovery (Cambridge, UK). Human immortalized astrocytes were generated as described below. All stable isotopes were purchased from Cambridge Isotope Laboratories (Tewksbury, MO). AGI-5198 was purchased from Cayman Chemical (Ann Arbor, MI). Palmitate-BSA conjugate was purchased from Seahorse Bioscience (Santa Clara, CA).

### Generation of *IDH1* Mutant Astrocytes

We serially transduced human fetal astrocytes (Sciencell Research Laboratories) with pBabe-hygro hTERT (Counter et al., 1998), pBabe-Neomycin-DD (dominant-negative allele of p53) (Hahn et al., 2002), and pMKO-puromycin p16 shRNA (Boehm et al., 2005), in that order. After each infection, cells were selected with 400 mg/mL hygromycin, 800 mg/mL G418, or 1.5 mg/mL puromycin. We cultured cells on poly-D-lysine or poly-L-lysine plates (BD Biosciences) in Astrocyte Media (Sciencell Research Laboratories).

IDH1-FLAG was cloned using restriction enzyme digestion from the pSLIK-IDH1-FLAG plasmid and inserted into the pMIG plasmid. The R132H mutation was then introduced into the pMIG IDH1-FLAG using the Q5 site-directed mutagenesis kit (NEB) according to manufacturer’s instructions. pSLIK-IDH1-FLAG was a gift from Christian Metallo (Addgene plasmid #66802), and pMIG was a gift from William Hahn (Addgene plasmid #9044). 8 × 10^5^ 293T cells were plated, and the next day retrovirus was generated by transfecting 9 mg of the pMIG IDH1^WT^ or pMIG IDH1^R132H^ plasmids with 1 mg of pCL Ampho packaging plasmid using Fugene 6 transfection reagent according to manufacturer’s instructions. Media containing the retrovirus was then collected and filtered using a 0.45-mm filter. 1 × 10^6^ immortalized human astrocytes (HAED16) were plated and the next day transduced with the pMIG IDH1^WT^ or pMIG IDH1^R132H^ retrovirus. GFP-positive cells were selected using FACS 72 hr later.

### Cell Culture and Sample Preparation

HCT116 cells were grown in McCoy’s 5A Modified Media (Thermo Fisher) with 10% FBS and no antibiotics, at 37 C and 5% CO_2_ (unless otherwise noted). Astrocytes were grown in Astrocyte Media with 2% FBS, 1% astrocyte growth supplement, and 1% penicillin-streptomycin (Sciencell). For all experiments, cells were plated at a density of 2.0 or 2.5 × 10^6^ cells per plate. Isotopic labeling experiments were performed in high-glucose DMEM, unless otherwise noted. Labeling experiments with 1,2-^13^C_2_ glucose included 2% FBS to minimize the concentration of unlabeled glucose. H_2_O_2_ experiments were conducted in media without FBS, as serum contains components that can act as a protectant against free radicals. Cells were harvested by aspirating media, then washing with PBS three times. This was followed by a wash with HPLC-grade water, upon which cells were then quenched with ice-cold HPLC-grade methanol. Cells were scraped from the plate and collected in methanol, pelleted, and dried via SpeedVac and subsequent lyophilization.

### Extraction of Metabolites

Cell pellets were extracted with methanol/acetonitrile/water (2:2:1), with solvent volumes normalized to a ratio of 1 mL of solvent per 1 mg of cell pellet. As previously described (Chen et al., 2016), samples were vortexed for 30 s and incubated for 1 min in liquid nitrogen, and then sonicated for 10 min. Following a 1-hr incubation at 20 C, the samples were centrifuged at 14,000 rpm for 10 min. The supernatant was collected and dried via SpeedVac and reconstituted in acetonitrile/water (1:1). The reconstitution solvent volume was normalized to a ratio of 100 mL per 1 mg of initial cell pellet.

### Determination of PPP Flux

Relative PPP flux was quantified by using 1,2-^13^C_2_ glucose as a tracer, as previously described (Lee et al., 1998; Li et al., 2014). For measuring relative PPP flux and PPP/glycolysis ratios, cells were grown for 24 hr in DMEM with 10% FBS. Media was then exchanged for DMEM containing 5 mM 1,2-^13^C_2_ glucose and 2% FBS for a 12-hr incubation before cells were harvested. The M+1/M+2 ratio indicates the ratio of glucose cycled through the oxidative PPP to glucose going directly through glycolysis. We determined PPP flux with the following formula: relative PPP flux = glucose uptake rate × [M+1 lactate/(M+2 lactate + M+1 lactate)]. Flux was normalized to wild-type cells by setting the wild-type value equal to 1.

### Measurement of Cell Viability

Cell viability was measured both with a trypan blue exclusion assay as well as a lactate dehydrogenase (LDH) cytotoxicity assay (Biovision) after exposure to H_2_O_2_. Cells were counted following the trypan blue exclusion assay. The LDH assay was performed in a 96-well plate, where 1.0 × 10^5^ cells per well in 200 mL of media were determined to be the optimal target cell number. Per the user manual, cells were exposed to H_2_O_2_ and LDH release was measured spectrophotometrically at 490 nm.

### LC/MS-Based Metabolomics

Metabolite analysis was performed on a Thermo Scientific Q Exactive Plus Orbitrap connected to a Dionex UltiMate HPLC system (Waltham, MA) in negative ionization mode with 70,000 resolving power. For each sample, 3 μL was injected onto a Luna Aminopropyl column (3 μm, 150 mm × 1.0 mm inner diameter [I.D.]; Phenomenex, Torrance, CA) set to a flow rate of 50 μL/min. Mobile phase A was 95% water, 5% acetonitrile (ACN), 20 mM ammonium hydroxide, and 20 mM ammonium acetate. Mobile phase B was 95% ACN and 5% water. The column was kept at 30 C for the duration of the following linear gradient: 0–45 min, 100%–0% B; 45–50 min, 0% B; 50–51 min, 0%–100% B; 51–60 min, 100% B (isocratic). Analysis of fatty acids was performed on an Agilent 6530 Q-TOF using an electrospray ionization (ESI) source with Agilent Jet Stream Technology. Mass range was set to 100–1,500 *m/z* in positive ionization mode. Aliquots of 2 μL of sample were injected onto a CORTECS UPLC T3 column (1.6 μm, 150 mm × 2.1 mm I.D.; Waters Corporation, Milford, MA) connected to an Agilent 1290 Infinity UHPLC system (Santa Clara, CA) with a flow rate of 200 μL/min. Mobile phase A was 100% water, 5 mM ammonium acetate, and 5 μM ammonium phosphate. Mobile phase B was 90% isopropanol, 10% methanol, 5 mM ammonium acetate, and 5 μM ammonium phosphate. The column was maintained at 55 C for the duration of the following linear gradient: 0–36 min, 0%–100% B; 36–40 min, 100% B (isocratic); 40–45 min, 100%–0% B.

### Metabolite Quantification

Glucose uptake and lactate secretion rates were determined by using an Agilent Q-TOF (6530) connected to an Agilent 1260 HPLC system (Santa Clara, CA) in negative ionization mode, as described above. 2-HG production flux was measured by quantitating media 2-HG levels over 24 hr. NADPH levels were measured with a NADP^+^/NADPH Quantification kit (BioVision) and a BioTek Cytation 5 plate reader (Winooski, VT).

### Radiation Exposure

Cells were pulsed with 0, 3, 6, and 9 Gy of IR by using a Precision X-Ray (North Bradford, CT) X-RAD 320 biological irradiator. HCT116 cell death was measured with a trypan blue exclusion assay 72 hr after radiation exposure. Astrocytes did not show significant cell death at 72 hr and were assayed at 96 hr with trypan blue.

### Statistical Analysis

All experiments were performed in triplicate (n = 3). All p values were calculated with a two-tailed Student’s paired t test.

## Supplementary Material

1

2

## Figures and Tables

**Table 1:** ISA Values from Cells Labeled with U-^13^C Glucose for 24 and 120 hr Show That the Fractional *De Novo* Synthesis of Palmitate Is Decreased in R132H/+ Cells

	WT^a^	R132H/+^a^	WT^b^	R132H/+^b^
*D*_glucose_	0.55 ± 0.04	0.54 ± 0.02	0.67 ± 0.01	0.68 ± 0.04
*g*	0.50 ± 0.05	0.41 ± 0.03	0.77 ± 0.03	0.59 ± 0.07

a 24-hr label time.

b 120-hr label time.

## Highlights

- Synthesis of 2-HG is supported by increased PPP activity
- Consuming NADPH for 2-HG synthesis contributes to an NADPH deficit
- 2-HG synthesis competes with other NADPH-requiring reactions
- Synthesis of 2-HG is regulated by NADPH availability
