# Supplementary material for: Consumption of NADPH for 2-HG Synthesis Increases Pentose Phosphate Pathway Flux and Sensitizes Cells to Oxidative Stress
Source: Cell Rep. Author manuscript; Available in PMC 2018 Jul 20. (PMC6053654; doi:10.1016/j.celrep.2017.12.050)
Supplement: 1 [file NIHMS935575-supplement-1.pdf]

Cell Reports, Volume *22*

Supplemental Information

Consumption of NADPH for 2-HG Synthesis Increases Pentose Phosphate Pathway Flux and Sensitizes Cells to Oxidative Stress

Susan J. Gelman, Fuad Naser, Nathaniel G. Mahieu, Lisa D. McKenzie, Gavin P. Dunn, Milan G. Chheda, and Gary J. Patti

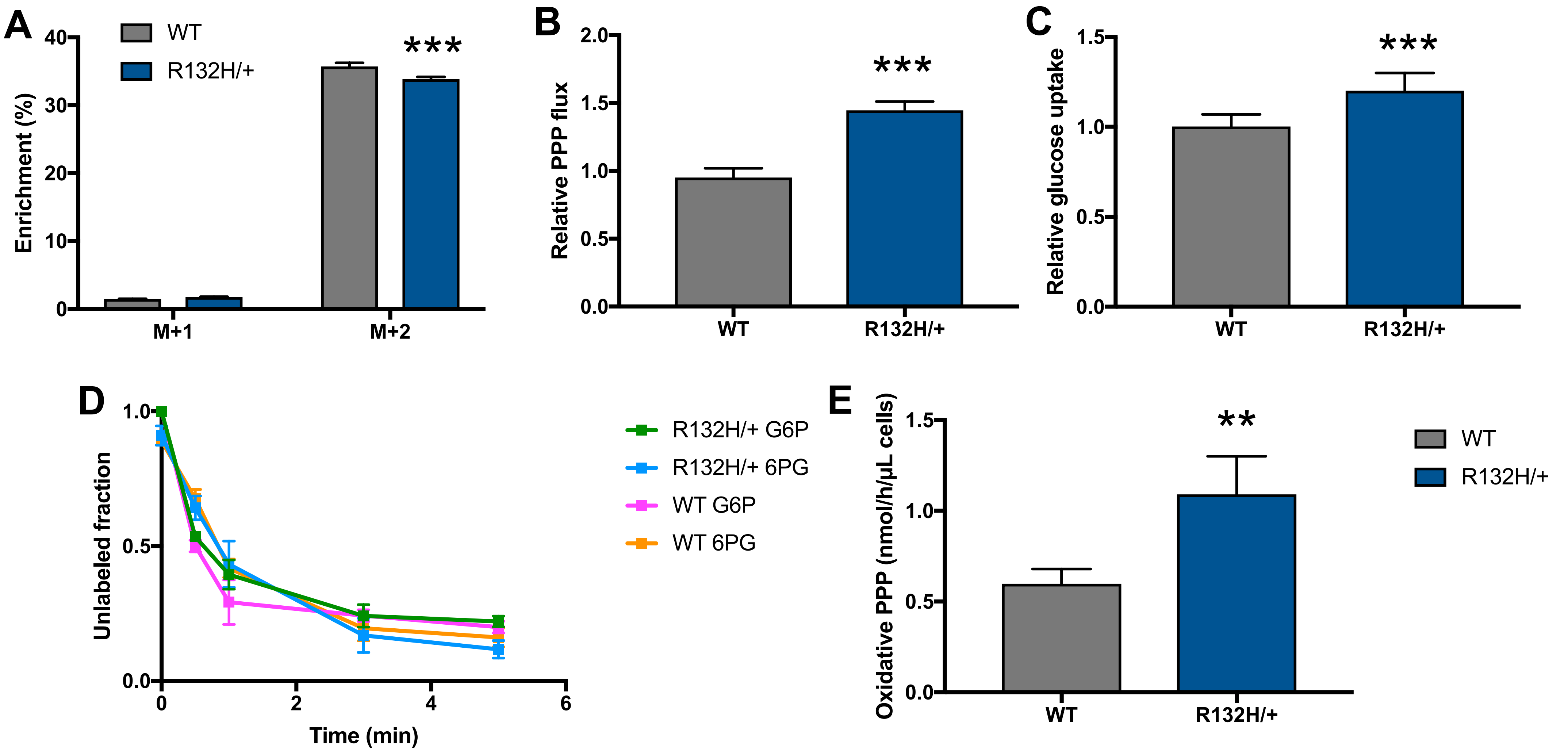

Figure S1. *IDH1* mutants have increased PPP flux, Related to Figure 3. (A-C) *IDH1* mutants have increased PPP flux when cultured in DMEM with 10% FBS and 5 mM glucose. (A) Isotopologue distribution of lactate in wild-type cells and HCT116 R132H/+ mutants. The M+1 isotopologue is a result of glucose carbon that passed through the PPP. The M+2 isotopologue corresponds to glucose carbon that was metabolized to lactate through glycolysis directly. (B) Relative PPP flux, as determined by lactate labeling from 1,2-$^{13}C_2$ glucose and glucose uptake. (C) Uptake of glucose by wildtype HCT116 cells and *IDH1* mutants. (D-E) The flux of the oxidative phase of the PPP is increased in HCT116 *IDH1* mutants relative to wild-type cells. (D) Decay of the unlabeled fraction of glucose 6-phosphate (G6P) and the unlabeled fraction of 6-phosphogluconate (6PG) in wild-type and R132H/+ cells. (E) Oxidative PPP flux was determined by fitting G6P and 6PG data with Newtonian type minimization, using the measured 6PG concentrations of 2.9 pmol per μL of cells and 3.5 pmol per μL of cells for wild-type and R132H/+ cells, respectively. Data shown are mean values ± s.d. (n=3). ** indicates a p-value < 0.01, and *** indicates a p-value < 0.001.

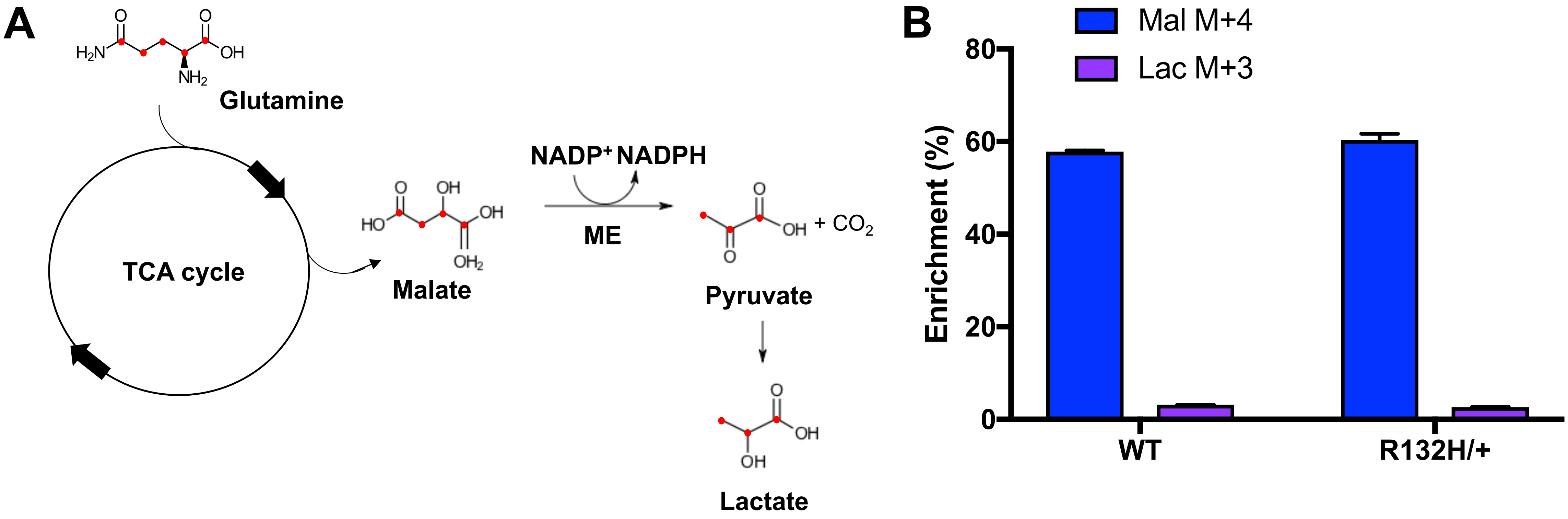

Figure S2. Evaluating the activity of malic enzyme (ME) with U-^{13}C glutamine, Related to Experimental Procedures. (A) Schematic to show labeling when U-^{13}C glutamine is transformed to lactate through ME. The ratios of labeled malate and lactate were analyzed. (B) Labeling of malate and lactate from U-^{13}C glutamine. The data do not support increased ME activity in *IDH1* mutants relative to wild-type cells. It is important to note that this method does not differentiate between ME subtypes or compartmentalization of the NADPH produced. Data shown are mean values ± s.d. (*n*=3).

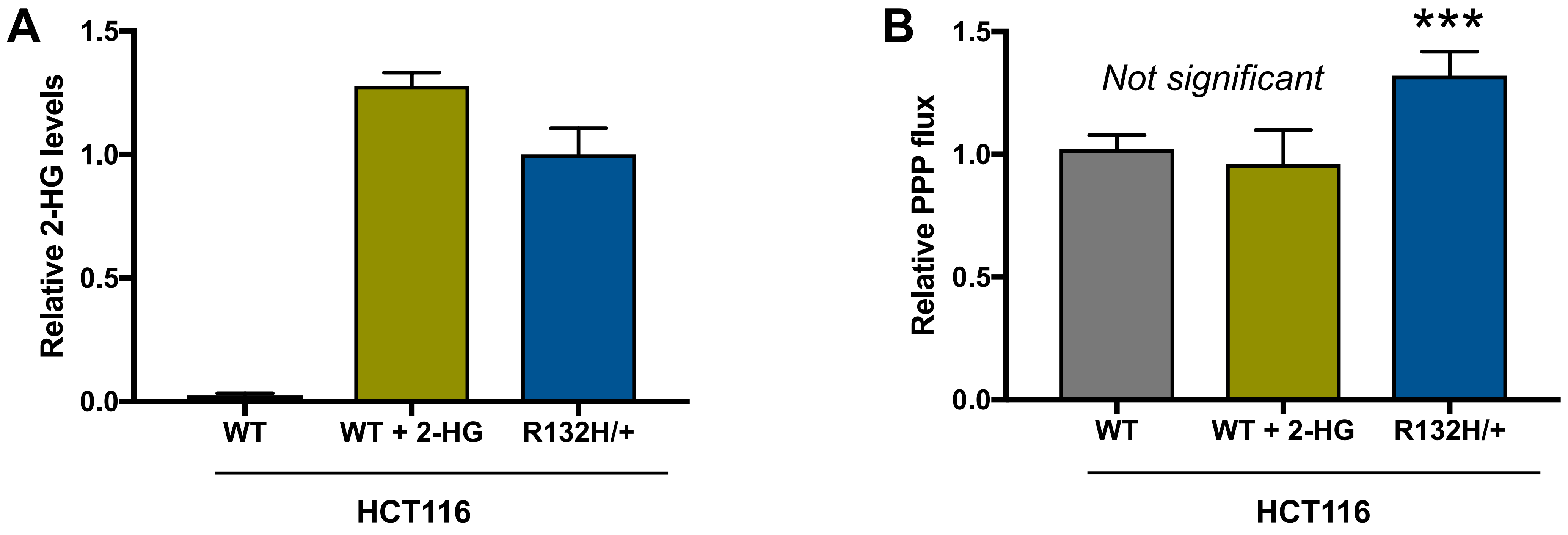

Figure S3. Exposing cells to 2-HG does not increase PPP flux, Related to Figure 3. (A) LC/MS measurements show that the intracellular concentration of 2-HG is comparable between HCT116 R132H/+ cells and wild-type cells treated with 0.1 mM octyl 2-HG. Wild-type cells alone display very low levels of 2-HG. (B) PPP flux of HCT116 wild-type cells exposed to 0.1 mM octyl 2-HG for 72 hrs. Compared to control wild-type cells, no significant change in PPP flux is detected upon treatment with octyl 2-HG. R132H/+ cells, however, have increased PPP flux relative to both wild-type and wild-type + octyl 2-HG. Data shown are mean values ± s.d. (n=3). *** indicates a p-value < 0.001.

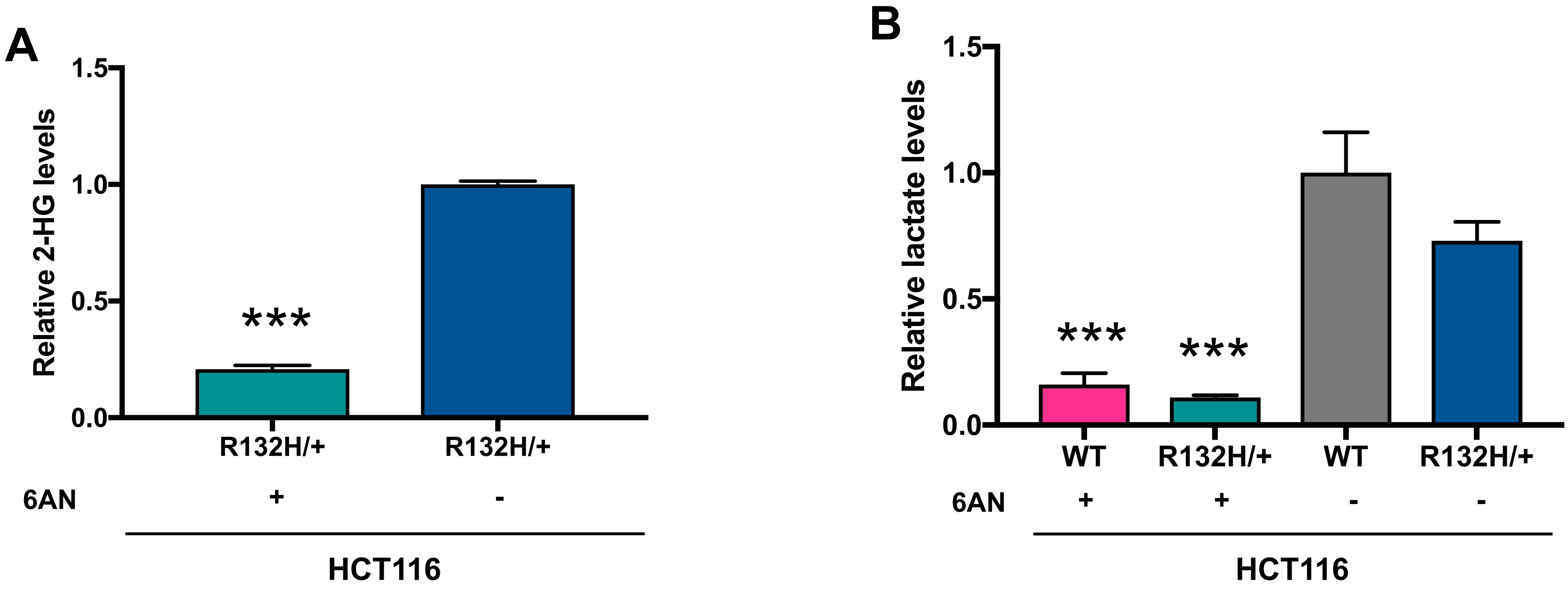

Figure S4. Effects of 6-aminonicotinamide, an inhibitor of 6-phosphogluconate dehydrogenase, Related to Figure 3. (A) Intracellular levels of 2-HG decrease after treating HCT116 R132H/+ cells with 6-aminonicotinamide for 24 hr. (B) The intracellular levels of other central carbon metabolites, such as lactate shown here, also decrease upon treatment with 6-aminonicotinamide for 24 hr. Data shown are mean values ± s.d. (*n*=3). *** indicates a *p*-value < 0.001.

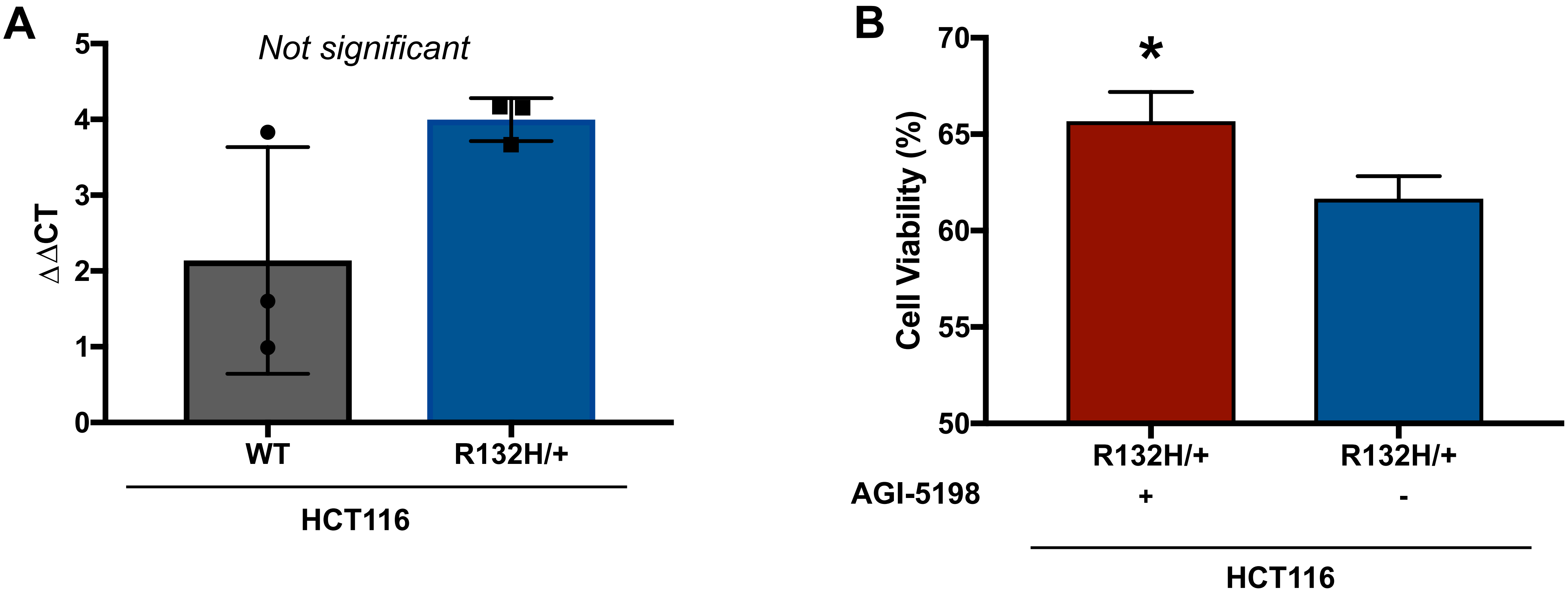

Figure S5. **Assessing the effects of increased NADPH consumption, Related to Experimental Procedures.** (A) Expression of G6PD is not statistically different between wildtype cells and *IDH1* mutants. ΔΔCT values were determined by qPCR using beta actin as a housekeeping gene. (B) AGI-5198 protects *IDH1* mutants during H_2O_2 exposure. Cells were exposed to 1 mM H_2O_2 and cell viability was measured with a trypan blue exclusion assay. Data shown are mean values ± s.d. (*n*=3). * indicates a *p*-value < 0.05.

Supplemental Tables

Supplemental Table S1. Fluxes of PPP and 2-HG production, Related to Figure 3.

	WT	R132H/+
**PPP flux (fmol per glucose per cell per hour)**	4.85 ± 0.17	6.92 ± 0.23
**2-HG production flux (fmol/cell/hr)**	-	6.50 ± 0.43

Supplemental Table S2. ISA values from cells labeled with U-^{13}C glucose in the presence of exogenous acetate show that R132H/+ mutants use more acetate for palmitate synthesis compared to wildtype cells, Related to Figure 5.

	WT acetate	R132H/+ acetate
$D_{glucose}$	0.62 ± 0.02	0.57 ± 0.02
g (24 hr)	0.68 ± 0.03	0.61 ± 0.04

Supplemental Experimental Procedures

Kinetic Flux Profiling

Kinetic flux profiling of the oxidative phase of the PPP was performed with U-^{13}C glucose. Both wildtype and R132H cells were exposed to labeled glucose for 0.5, 1, 3, and 5 min. Media was then aspirated and cells were immediately quenched with 80:20 methanol:water at 4 ºC. Cells were extracted as previously described (Yuan et al., 2008). The decay function of the unlabeled glycolytic and pentose phosphate intermediates were fitted with Newtonian minimizations and 95% confidence intervals were determined from t-values (OriginLab).

Quantitative RT-PCR

Total RNA was isolated with TRIzol Reagent (Invitrogen) and single-strand cDNA was synthesized with a First Strand Synthesis Kit (Origene). PCR was performed with PowerUp SYBR Green Master Mix (Thermo Fisher) and StepOnePlus PCR instrument at 60 ˚C.

Malic enzyme measurement

Cells were given U-^{13}C glutamine for 24 hr to assess malic enzyme activity. The M+3 isotopologue of lactate and the M+4 isotopologue of malate were used to evaluate malic enzyme activity (Fan et al., 2014).
